# Factors influencing deliveries at health facilities in a rural Maasai Community in Magadi sub-County, Kenya

**DOI:** 10.1186/s12884-017-1632-x

**Published:** 2018-01-03

**Authors:** Sarah Karanja, Richard Gichuki, Patrick Igunza, Samuel Muhula, Peter Ofware, Josephine Lesiamon, Lepantas Leshore, Lenny Bazira Kyomuhangi-Igbodipe, Josephat Nyagero, Nancy Binkin, David Ojakaa

**Affiliations:** 1 Amref Health Africa in Kenya, P.O. Box 30125-00100, Nairobi, Kenya; 2 Formerly Amref Health Africa, P.O. Box 27691-00506, Nairobi, Kenya; 3 Amref Health Africa, P.O. Box 27691-00506, Nairobi, Kenya; 4 University of California, 9500 Gilman Dr, La Jolla, San Diego, CA 92093 USA; 5 BRIM Consulting, P.O. Box 76100-00508, Nairobi, Kenya

**Keywords:** Health facility delivery, Skilled birth attendance, Maternal, Newborn and child health (MNCH), Traditional birth attendants (TBAs), Place of delivery

## Abstract

**Background:**

In response to poor maternal, newborn, and child health indicators in Magadi sub-county, the “Boma” model was launched to promote health facility delivery by establishing community health units and training community health volunteers (CHVs) and traditional birth attendants (TBAs) as safe motherhood promoters. As a result, health facility delivery increased from 14% to 24%, still considerably below the national average (61%). We therefore conducted this study to determine factors influencing health facility delivery and describe barriers and motivators to the same.

**Methods:**

A mixed methods cross-sectional study involving a survey with 200 women who had delivered in the last 24 months, 3 focus group discussions with health providers, chiefs and CHVs and 26 in-depth interviews with mothers, key decision influencers and TBAs. Adjusted odds ratios (aOR) and 95% confidence intervals (CI) using logistic regression were calculated to identify predictive factors for health facility delivery. Thematic analysis was done to describe barriers and motivators to the same.

**Results:**

Of the women interviewed, 39% delivered at the health facility. Factors positively associated with health facility deliveries included belonging to the highest wealth quintiles [aOR 4.9 (95%CI 1.5–16.5)], currently not married [aOR 2.4 (95%CI 1.1–5.4)] and living near the health facility [aOR 2.2 (95%CI 1.1 = 4.4)]. High parity [aOR 0.7 (95%CI 0.5–0.9)] was negatively associated with health facility delivery. Barriers to health facility delivery included women not being final decision makers on place of birth, lack of a birth plan, gender of health provider, unfamiliar birthing position, disrespect and/or abuse, distance, attitude of health providers and lack of essential drugs and supplies. Motivators included proximity to health facility, mother’s health condition, integration of TBAs into the health system, and health education/advice received.

**Conclusion:**

Belonging to the highest wealth quintile, currently not married and living near a health facility were positively associated with health facility delivery. Gender inequity and cultural practices such as lack of birth preparedness should be addressed. Transport mechanisms need to be established to avoid delay in reaching a health facility. The health systems also need to be functional with adequate supplies and motivated staff.

**Electronic supplementary material:**

The online version of this article (10.1186/s12884-017-1632-x) contains supplementary material, which is available to authorized users.

## Background

The third sustainable development goal calls for a reduction of the global maternal mortality ratio to less than 70 per 100,000 live births, neonatal mortality to at least as low as 12 per 1000 live births and under-5 mortality to at least as low as 25 per 1000 live births by 2030 [1]. Although the number of deaths in children under five declined worldwide from 12.7 million in 1990 to 5.9 million in 2015, an increasing proportion of these deaths occur in sub-Saharan Africa, where 1 child in 12 dies before his or her fifth birthday [2]. Low and middle-income countries accounted for 99% of all maternal deaths in 2015 and sub-Saharan Africa for 66% [3]. Such inequities occur not only between high income and low and middle-income countries, but also within countries, where maternal and child mortality rates differ by geographic location as well as social position and ownership or control of resources.

A number of high-impact interventions to reduce maternal, newborn and child mortality have been identified, of which skilled birth attendance is one of the most effective [4]. Delivering in a health facility provides a key window of opportunity to receive high impact interventions during the delivery itself, including management of eclampsia and hemorrhage, neonatal resuscitation, support for early initiation and exclusive breast feeding support, and immediate treatment of suspected infection [5].

In many sub-Saharan African countries, levels of skilled birth attendance are low. The lack of skilled health personnel during pregnancy and delivery largely account for an estimated 289,000 mothers and 2.8 million newborn annual deaths in the first month of life [6]. In Kenya, only about 62% of births are delivered by skilled health personnel despite concerted efforts by the government to increase skilled birth attendance [7].

The government of Kenya’s interest in maternal, newborn and child health has been intense, and several official documents capture this emphasis. These include “The Six Pillars of Kenya’s MNCH Programme” and the “Strategic Framework for the Engagement of the First Lady in HIV Control and Promotion of Maternal, Newborn and Child Health in Kenya” [8]. One of the outcomes of this interest is that all delivery services in public facilities are now free of charge, thus eliminating some of the financial barriers to access.

An important issue in the ongoing implementation of Maternal Newborn Child Health (MNCH) programmes under these frameworks has revolved around the assumption that mass implementation of MNCH programmes means that all communities in a given intervention area have access and benefit from the services. This is an important issue in Kenya because many marginalized communities such as pastoralists continue to live in remote areas where access to formal health services is limited [9].

Using both quantitative and qualitative methods, the objective of this study was to determine which factors influence the utilisation of health facility delivery services among Maasai women in the Entasopia community of Magadi County in southern Kenya. More specifically, a quantitative study was conducted to document the frequency of facility-based deliveries, socioeconomic factors associated with deliveries, and the extent to which access to these services was equitably distributed in the community. The qualitative component sought to describe the reasons women do or do not deliver in the health facilities under skilled birth attendants. The study was conducted within a sub-county which had been part of the *“Boma”* model, an intervention that Amref Health Africa in Kenya implemented initially in the Magadi area but which has expanded to the other Maa-speaking county of Samburu in northern Kenya.

### The *“Bom*a” model intervention

The essence of the *“Boma”* model is that it recognizes the Maasai homestead, which is known as a *“Boma”* in Swahili, as the ideal interface between the semi-migratory community and MNCH health care delivery. It focuses on developing linkages between the community (level 1 of the health care system) and the formal health care system, particularly level two (dispensary), level three (health centre) and level four (county hospital) of the health care system. Project objectives included 1) to increase utilization of MNCH services provided by skilled health professionals in line with ministry of health guidelines; 2) to improve case management of childhood illnesses at community and health facility; and 3) to strengthen the community health information system and link it to the health facility information system.

The *“Boma”* model achieved a number of significant successes in increasing utilization of MNCH services provided by skilled health professionals in line with guidelines from the Ministry of Health. The proportion of pregnant women who attended four ANC visits increased from 4% in 2007 to 32% in 2010 and 59% by 2012 [10]. The 2012 end-term evaluation for the project revealed that more mothers were initiating ANC early in the first trimester (29% compared to 22% at baseline) [10]. Skilled attendant deliveries improved from 14% to 24%; however, this achievement is below the national average of 61% [7].

## Methods

### Study design

This was a cross sectional study using mixed methods approach to assess factors that influence health facility delivery. Quantitative research methods were used to assess coverage of facility births by various equity-related characteristics, while qualitative research methods identified motivators and barriers to health facility delivery.

### Study setting

This study was conducted in Entasopia community, which is located in Magadi sub-county of Kajiado County, Kenya. The area is one of the arid and semi-arid lands (ASALs) in Kenya, which are classified by the Kenya government as disadvantaged with respect to equitable distribution of national resources, infrastructure and access to essential social services including healthcare. ASAL regions are home to nomadic and semi-nomadic communities such as the Maasai and Turkana who have poor MNCH indicators. The poor MNCH indicators among these communities are attributed to a complexity of factors including inadequate, ill-equipped and poorly staffed health facilities; long distances to health facilities; migratory lifestyles; conservative cultural practices; and gender biases [10]. Magadi County was chosen as a study location because initially the *“Boma”* model was implemented there prior to extending it to Samburu County. The county has eight community units (Oldonyonkie, Shompole, Olkeri, Olkiramatian, Oloika, Pakase, and Entasopia, which contains two community units). Entasopia was the only community unit that had a relatively better quality health facility with 24-h obstetric care, and since the purpose of the case study was to understand why some women use and others do not use health facilities for delivery beyond lack of geographic access, it was decided to limit the case study to this unit. Data was collected from September 22 to October 3, 2014.

## Quantitative methods

### Participants

Households were used as the unit of selection. All households within community health units in the study site were mapped and given a unique number which gave rise to sampling frame. Systematic sampling technique was used to sample 50% of the total households. In the sampled households, all women aged between18–49 years and had given birth in the last 24 months were interviewed.

### Variables

The outcome variable of interest was the dichotomous variable most recent delivery in a health facility (yes/no). Alternatives to using a health facility for delivery included delivered at home or on the way to the health facility. The explanatory variables included the women’s self-reported education level, age, religion, marital status, occupation, number of antenatal care visits, distance between home and the facility, number of children in the household and household possessions used to construct wealth quintiles. We generated a list of indicator variables using Demographic and Health Surveys (DHS) with the assistance of staff and CHVs who implemented the *“Boma”* project. One indicator variable (i.e. total number of livestock) that is not in DHS list but is a symbol of wealth in Maasai community was included.

### Sample size

Fischer’s formula which is the standard formula for cross-sectional studies was used to establish the sample size. Assuming 95% confidence level, ±5% precision and 50% prevalence, a sample size of 385 was estimated. This formula assumes that the target population is more than 10,000 and recommend sample size adjustment if target population is less than 10,000. Since the study target population was less than 10,000, the sample size was adjusted using the standard method to 257 participants. During the analysis, Maasai speaking participants (*n* = 200) were considered.

### Data collection

A survey tool was administered to women who had delivered their last child in the last 24 months (Additional file 1). Data were collected on paper forms and entered into two electronic spreadsheets using Microsoft Access. Ten percent of the questionnaires were randomly selected and rechecked for correct entry against the two spreadsheets, and the spreadsheet that had the lowest error percentage (1.8%) was selected for final analysis. Prior to the analysis, further data cleaning was carried out using frequency distributions and cross-tabulations of the dataset. The data were also coded and the required composite variables created. Finally, all personal identifiable characteristics such as name and date of birth were removed from the database. All analyses were conducted using SPSS version 20.

### Quantitative data analysis

Five quantitative variables were involved; age, parity, antenatal care visits, distance from the health facility and wealth index. Antenatal care visits and distance from the health facility variables were each grouped into two categories (Antenatal care visits; fewer than four/ four or more, distance from the health facility; less than 3kms/ more than 3kms). A wealth index was developed and grouped into wealth quintiles (lowest, low, medium, high and highest). Wealth quintiles were calculated with standard techniques based on principal component analysis on the reported ownership of household possessions, main sources of income, lighting, and cooking fuel. Characteristics that did not show variation across households (e.g. ownership of mobile phones or type of toilets) were not included in principal component analysis. The variables included in the development of the wealth quintiles for the analysis included: total livestock, head of household occupation, woman occupation, ownership of (bicycle, motorcycle, radio, TV, foam mattress, gas cooker, furniture, solar, other). Variables with very few respondents (e.g. ownership of car, refrigerator and sewing machine), were merged to ‘other’ category. Age and parity were not grouped during analysis.

Potential predictive factors for facility delivery were examined initially using bivariate analysis, followed by binary logistic regression with SPSS version 20. In bivariate analyses, statistical tests which include Chi-square test and t-test were used if assumptions associated with their use had not been violated. Where assumptions were not met, Fisher’s exact test was used instead of Chi-square and non-parametric tests used instead of t-test. Statistical significance was set at *p*-value <0.05. Independent variables were used in the binary logistic regression model if they were either statistically significant in bivariate analysis or if they had been demonstrated elsewhere to be significant predictors of delivering in a health facility.

## Qualitative methods

### Participants

As the target subjects of the *“Boma”* model interventions, we considered women who had delivered their youngest child in the last 2 years. We also sought the perspectives of homestead heads as they had been targeted by the *“Boma”* model as entry points to influence decision making on health issues within the household. CHVs, the provincial administrators (chiefs), health care workers, village elders and TBAs also played a pivotal role in the implementation of the *“Boma”* model either as links to the health facility or service providers, and their views were also explored.

### Sample size

Two study villages, one within a short walking distance to the health centre and therefore with optimal access and one approximately more than 5 km away but where access to the health facility was still possible, were chosen for the qualitative portion of the study. Within each village, four women who delivered in a health facility and four women of similar parity who delivered at home were interviewed using a semi-structured in-depth interview guide. The key decision-influencers of women, that is, husband/mother/mother-in-law, for two women who delivered in a health facility and two who did not were also interviewed to better understand the decision-making process. In addition, the village elder and the traditional birth attendants in each village were interviewed. Finally, three focus group discussions with CHVs, the provincial administrators (Chiefs), and health care workers were conducted.

The maternity register at the Entasopia Health Center was used to identify women residing in the two villages and who had delivered there within the last 2 years. CHVs attached to the respective villages guided the interviewers to the “*Bomas”* of the identified women, as well as to the Bomas of women who had delivered at home. Since the CHVs were well known by the respondents, they assisted in introducing the research assistants who then introduced the study and sought informed consent prior to conducting the interviews. Table 1 outlines the type and number of respondents interviewed and the qualitative data collection method used.

**Table 1:** Description of qualitative data collected

Participant	Number of participants
IDIs in a village far away from the health facility	
Women who recently delivered their first born at home	2
Women who recently delivered their first born in a health facility	2
Women who recently delivered their 2nd born or above baby in a health facility	2
Women who recently delivered their 2nd born or above baby at home	2
Village elders	1
Key decision influencer for a woman who delivered at home	1
Key decision influencer for a woman who delivered in a health facility	1
TBA who conducts deliveries at home	1
TBA who escorts women to deliver in a health facility	1
TOTAL	**13**
IDIs in a village near the health facility	
Women who recently delivered their first born at home	2
Women who recently delivered their first born in a health facility	2
Women who recently delivered their 2nd born or above baby in a health facility	2
Women who recently delivered their 2nd born or above baby at home	2
Village elders	1
Key decision influencer for a woman who delivered at home	1
Key decision influencer for a woman who delivered in a health facility	1
TBA who conducts deliveries at home	1
TBA who escorts women to deliver in a health facility	1
TOTAL	**13**
FGDs	
Chiefs	8
Heath Care Workers	8
Community Health Workers	12
TOTAL	**28**

### Data collection

Four research assistants who carried out the interviews underwent four-day training in conducting in-depth interviews and focus group discussions, including note taking, debriefing, transcribing and translating (Additional files 2, 3, 4, 5, 6, 7, 8 and 9). They all spoke the local Maasai dialect and used interview guides translated into Maasai to carry out the interviews. All interviews were tape-recorded and at the end of each day, the recordings were transferred to a password-protected study computer and backed up on an external hard drive. Debriefing sessions to reflect on each interview were held at the end of each day and written on a debriefing template. Debriefing notes and notes taken during the interviews were typed out and saved on the study computer. All interviews were transcribed verbatim and translated into English where necessary.

### Qualitative data analysis

Following transcription of all interviews data were analyzed using a thematic analysis approach by two researchers (SK and PI) [11]. In the development of themes we adopted both a deductive and inductive approach. In the initial analysis a deductive approach was used to identify pre-set themes from the interview guides (topic guides). Transcripts were then read extensively by SK and PI using an inductive approach to identify emerging themes from the data. The emerging themes were those ideas or concepts that came up in the data and were different from the pre-set themes. SK coded all transcripts and PI coded a sub-set of the transcripts (approximately 20%) to ensure inter-coder reliability. SK and PI discussed any inconsistencies until mutual agreement was reached. The relationship between the quotes and codes was then explored to answer the research questions on motivators and barriers to health facility delivery. NVivo 10 software was used by both researchers throughout the analysis process.

## Results

### Quantitative results

Of the 200 Maasai-speaking women whose data were analyzed, mean age was 25.8 years, 137 (69%) had no formal education and 78 (39%) delivered in a health facility. Table 2 presents the socio-demographic characteristics of women by place of delivery and factors influencing health facility delivery. At bivariate analysis, level of education (*p* < 0.001), age (*p* = 0.05), marital status (*p* = 0.001), occupation (*p* = 0.015), parity (p < 0.001) and wealth quintiles (p = 0.001) were significantly associated with health facility delivery. By contrast, neither religion nor ANC was significantly associated with health facility delivery. After adjusting for other factors, belonging to the highest wealth quintile [aOR 4.9 (95% CI 1.5, 16.5)], currently not married [aOR 2.4 (95% CI 1.1, 5.4)], and living near the health facility [aOR 2.2 (95% CI 1.1, 4.4)] were positively associated with health facility delivery. High parity [aOR 0.7 (95% CI 0.5, 0.9)] was negatively associated with health facility delivery.

**Table 2:** Bivariate and multivariate analysis of the association between socio-demographic characteristics and health facility delivery

Characteristics	Total n (%)	Health facility delivery		Unadjusted OR (95% CI)	Adjusted OR (95% CI)
		Yes n (%)	No n (%)		
Total sample	200 (100%)	78 (39%)	122 (61%)		
Education Level					
None (Ref)	137 (68.5%)	38 (27.7%)	99 (72.3%)	1	1
Primary	50 (25.0%)	30 (60.0%)	20 (40.0%)	4.0 (2.0, 7.7)	1.8 (0.8, 4.01)
Post Primary	13 (6.5%)	10 (76.9%)	3 (23.1%)	8.69 (2.3, 33.3)	2.3 (0.5, 11.3)
Age (Mean(SD))	25.8 (7.3)	24.5 (6.1)	27.0 (7.7)	0.94 (0.90, 0.98)	1.0 (0.9, 1.1)
Marital status					
Married Polygamous (Ref)	73 (36.5%)	17 (23.3%)	56 (76.7%)	1	1
Married Monogamous	104 (52.0%)	47 (45.2%)	57 (54.8%)	2.72 (1.4, 5.3)	2.8 (0.7, 10.1)
Currently not married	23 (11.5%)	14 (60.9%)	9 (39.1%)	5.12 (1.9, 13.9)	2.4 (1.1, 5.4)
Religion					
Others (Ref)	40 (25.0%)	15 (37.5%)	25 (62.5%)	1	
Christians	160 (75.0%)	63 (39.4%)	97 (60.6%)	1.08 (0.5, 2.2)	
Occupation					
No income (Ref)	154 (77.0%)	53 (34.4%)	101 (65.6%)	1	1
Earns income	46 (33.3%)	25 (54.3%)	21 (45.7%)	2.27 (1.2, 4.4)	1.7 (0.7, 4.1)
No. of children (Median (Range)	3 (1,8)	2 (1,6)	3 (1,8)	0.66 (0.5, 0.8)	0.7 (0.5, 0.9)
ANC visits					
less than 4 visits (Ref)	35 (17.5%)	11 (31.4%)	24 (68.6%)	1	
4+ visits	162 (82.5%)	66 (40.7%)	96 (59.3%)	1.50 (0.7, 3.3)	
Distance					
Far (>3 km) (Ref)	96 (48.0%)	26 (27.1%)	70 (72.9%)	1	1
Near (≤3 km)	104 (52.0%)	52 (50.0%)	52 (50.0%)	2.69 (1.5, 4.9)	2.2 (1.1, 4.4)
Wealth quintiles					
Lowest (Ref)	40 (20.0%)	8 (20.0%)	32 (80.0%)	1	1
Low	40 (20.0%)	9 (22.5%)	31 (77.5%)	1.16 (0.4, 3.4)	1.4 (0.4, 4.5)
Medium	40 (20.0%)	18 (45.0%)	22 (55.0%)	3.27 (1.2, 8.8)	4.5 (1.5, 14.1)
High	40 (20.0%)	19 (47.5%)	21 (52.5%)	3.62 (1.3, 9.8)	4.9 (1.5, 15.5)
Highest	40 (20.0%)	24 (60.0%)	16 (40.0%)	6.0 (2.2, 16.3)	4.9 (1.5, 16.5)

### Qualitative results

These results are organized into barriers and motivators to health facility delivery.

### Barriers to health facility delivery

There are three groups of factors that appeared to hinder health facility delivery: Individual/family/community factors; geographic factors and health facility factors.

### Individual/family/community factors

It appeared that most women were not the final decision-makers on the place of birth. The head of the household who is the eldest person in the *“Boma”* had the ultimate decision-making power. For example:

*“…as the head of the “Boma” I must always give a go ahead of anything that need to be done at my home so generally the mother can suggest but I am the final decision maker”* (FGD with CHVs)

“*…she is mine and I will say where she will give birth” -* Key decision-influencer for a woman who recently delivered in a health facility.

Most participants mentioned they did not make any birth plans when they discovered they were pregnant since in the Maasai community giving birth is seen as a natural occurrence and it is a taboo to make any birth plans.

*“According to Maasai, you can’t choose for the baby the place you want to deliver; you just wait for the event to happen because the child can just come out at any place so there is no issue of planning.”* A woman who delivered at home from a village far from the health facility

As a result, the decision on place of birth was made at the onset of labor unless a woman had previous health complications or had previously undergone a caesarean operation. Women were more likely to deliver at home if they had previously had a home delivery without experiencing any complications, thereby viewing facility-based delivery as unnecessary.

*“It is because I have delivered all my children there (home), even this one because the TBA who assisted me is trained by Amref and so it is equal to a hospital delivery*.” - A woman who lives near a hospital but delivered at home

Misconceptions about the services provided at the health facility, as well as the perceived quality of care also hindered women from delivering at the health facility. Participants believed that in some health facilities, women undergo unnecessary cesarean section and thus women avoid giving birth in these facilities. For example:

“*Right now in…hospital, seven women were told that they will have to undergo operation because they could not deliver in the normal way. Those women came and delivered at home without any complications. Therefore that advice is frightening women*”. - Key decision influencer

The gender of the health care providers also played an important role in influencing the location of birth, as some women reported not being comfortable being attended to by a male nurse. For example:

*“When you turn to social factors, sometimes women don’t want to go to hospital because the attendant isn’t a woman, and don’t want to be attended to by a male, because she may not want everybody to see her private parts”-* FGD with chiefs

Some women prefer home delivery due to subjection to unfamiliar birthing position, such as lying on the back compared to squatting. Home deliveries for such women ensure they retain desired birth practices.

*“Others prefer home delivery because they say we don’t want to be told to lie on the back because it is uncomfortable. So such traditions matter”. –* TBA who delivers women at home

Separate focus group discussions with CHVs and chiefs revealed that women may not deliver at health facility due to fear of disrespect and/or abuse by health care providers. Verbal abuse in form of shouting or rudeness, and physical abuse in form of pinching were some of the examples cited.

*“…all the women would be delivering at hospital, it’s the issue of knowing who is the attendant at the hospital, may be Mr. (name) is the attendant and people know him as a rude person, so people opt elsewhere” –* FGD with Chiefs

*“There was another one who said that the reason why she doesn’t like going to deliver in the hospital is because there was a day when she was giving birth and a nurse pinched her with a pair of scissor so that she can push the baby, that was very painful to her and that discouraged her from delivering at the hospital.” -* FGD with CHVs

It appeared that some health care providers are known to be good and others have bad attitude towards the mothers.

*“They are not all the same, some are good and others are not…They treat people in a good and polite way but those who are not good shout at patients and do not show respect to them.”* - A woman who lives near the health facility and delivered at the health facility

### Geographic factors

Accessibility can be a barrier to health facility delivery due to geographical distance to the health facility, availability of means of transport, and poor terrain that increases the time taken to reach the health facility and thus causing the fear of delivering on the way to the hospital. For example:

*“Those who come from far have more challenges than others. There are places that even the ambulance can’t access…they have to be carried on motorbikes which are very risky for a mother in labor” -* FGD with heath care providers

*“Due to the distance to the health facility women are forced to deliver at home.” -* IDI with TBA who delivers women at home in a village far from the health facility

Apart from health facilities being far, maternity services are not provided at night at some facilities, hence when a woman goes into labor at night she might prefer to obtain the services of a TBA whether or not she lives near a health facility.

*“I didn’t choose I went into labor at night and who is at the hospital at night?”-* Woman from a village near a health facility but delivered at home

In an attempt to overcome such barriers, some participants who live far from the health facility relocated and stayed with relatives who lived near a health facility or used other means of transport such as motorbikes which were deemed much better compared to walking to a health facility.

### Health facility factors

Delay in receiving appropriate care due to negative attitude of health workers and lack of essential drugs and supplies was cited as a hindrance to health facility delivery. A key decision influencer for a woman who lives near a health facility but delivered at home also stated:

“*In our hospital, doctors neglect mothers a lot. You may call for assistance till the mother gives birth alone and she is in hospital.”*

In some cases the local administration had to be involved for health workers to provide maternity services at night.

*“The doctors now serve as they wish; there is no working at night nowadays unlike before. It is now a difficult issue. A chief has to be called to convince a doctor to attend to a patient…”* FGD with Chiefs

A CHV gave an example of unskilled birth attendance at the hospital when she escorted her daughter to deliver at the health facility.

*“There was a certain day when I brought my daughter here to deliver but the nurse refused because there were no gloves so I had to assist her myself.”*

### Motivators to facility based delivery

There are three groups of factors that appeared to motivate women to deliver in the health facility: individual, health system and geographic factors.

### Individual factors

Individual factors include the status of the woman’s health or birth experience in choosing the health facility as the birth location.

Having an existing health condition, such as high blood pressure, iron deficiency, or generally being sick, played a big role in motivating them to deliver in a health facility.

*“I think that’s what (health status) my husband considered because I became very sick and the nurses said that my blood level went down*” – Woman who lived near a health facility and delivered in a health facility

Women who previously planned to deliver at home, but who experienced prolonged labor were also likely to deliver in a health facility. A woman who lived near a health facility and had planned to deliver at home gives this example:

*“It was just because I had prolonged labor otherwise I could have given birth at home.”*

Some women preferred to deliver in the health facility because they were “used to giving birth there”.

### Geographic factors

Physical factors include the role of geographic distance in selection of a birth location; living near a health facility increased the likelihood of utilizing a health facility during delivery. Ease of walking to a health facility without having to worry about availability of transport was described as a facilitating factor.

*“I knew the hospital is close by so I would just go.”* – Woman who lives near a health facility and delivered in a health facility

### Health system factors

These include statements about the quality of care, availability of drugs and other commodities, and working closely with TBAs.

Availability of skilled personnel who can conduct life-saving interventions such as caesarean section, blood transfusion, labor induction and infusion was considered as a reason to deliver in a health facility.

*“At the hospital one can be helped in two ways because sometimes a woman is unable to deliver in the usual way and if the hospital is unable to help her deliver, she will be operated on and the baby removed, and once she delivers the doctors are close so that they serve her in any way necessary for the person’s health.”-* Key decision influencer

Availability of birth notification, drugs and other commodities given to women after delivering, such as diapers, towels, basins and mosquito nets, also motivated women to deliver in a health facility.

***“****When you give birth at the hospital…for that child who has been born, he/she has that document (birth notification) that day he/she is discharged. That is one advantage of giving birth at the hospital.” –* Key decision influencer

*“After I delivered, the placenta got retained and the nurses pressed my stomach, gave me two injections and it came out. So this was good because if I were at home I couldn’t have received this good care.”* Woman who lives far from the hospital but delivered in a health facility

TBAs that have been integrated into the existing health system accompanied women to the health facility, assisted midwives in comforting women during labor, and helped reduce the language barrier between the health workers and Maasai women. During the focus group discussion with health workers, the example below was given:

*“In this community, I think they have lived for long with the TBAs such that they have come to like them as part of them. So you never get a mother who comes to deliver without a TBA. So when you go to the maternity ward with that TBA, that mother will be very comfortable and won’t fear anything because the TBA is continually talking to them and instructing them on what to do. But when you go alone and leave the TBA outside you will get a hard time because there could be a communication barrier. You could be talking to them but they don’t get it, so unless they are there (TBAs) that is when you get the best delivery.”*

Health education/advice received during antenatal clinics, home visits or seminars encouraged women to deliver in a health facility.

*“We teach them the danger signs of pregnancies that they should be observing during pregnancy; we also tell them the importance of hospital delivery, and to give them nets and teach them how to use them for prevention of malaria during pregnancy.”* – FGD with health workers

*“I was the one who said let us go to hospital because whenever we go for clinic (ANC visit) we are usually advised that when we start to feel labor we should go to hospital”* - Woman who lived far from a health facility and delivered in a health facility

In some cases women would describe health facility delivery as a “rule” that had been put in place by the health care providers whereby they were compelled to follow it.

## Discussion

In this study, health facility delivery was 39% (78), which is still below the national average of 61% [12]. In the multivariate analysis, three factors were positively associated with health facility delivery with the most prominent factor being belonging to the highest wealth quintile, followed by currently not married and living near the health facility. High parity was negatively associated with health facility delivery.

With respect to reasons for not delivering in a facility, our study was conducted in an area that received an intervention to improve skilled birth attendance among other MNCH outcomes hence all participants were aware of the availability of skilled birth attendance. Despite this, the findings in this study suggest that most women did not deem it necessary to discuss the place of birth well in advance before the onset of labor unless a woman had a known pregnancy-related complication because it is a taboo to develop any birth plans since giving birth is considered a natural occurrence among the Maasai community. Another cultural practice that may have contributed to sub-optimal uptake of skilled birth attendance is that in this community women are not the final decision makers on the place of birth. Key decision influencers in the family such as father in law and husband are the main decision makers. There is need a to address cultural factors affecting skilled birth attendance and MNCH projects being implemented in such marginalized communities should take into consideration such factors by using participatory approaches to design innovations that can improve access to skilled birth delivery.

Additional factors influencing health facility delivery include fear of unnecessary cesarean section operation, gender of the healthcare provider, unfamiliar birthing position and fear of disrespect and/or abuse. These findings are similar to other studies and have been shown to contribute to poor uptake of skilled birth attendance [13–15]. On the other hand, they provide an opportunity for improvement of the quality of care provided during delivery.

Distance to the health facility was a significant determinant of health facility delivery, with those living within a 3 km radius to the health facility having a 2-fold higher likelihood of delivering in a facility. Our findings suggested confirmation from other studies that lack of appropriate means of transport may limit women from physically accessing health facility during labor [16]. Some participants in our study overcame the physical barriers to access to a health facility by relocating and staying with relatives who lived near a health facility. Maternity waiting homes can also be built near health facilities thus providing women with various options to overcoming distance to the health facility. Though there is insufficient evidence on effects of maternity waiting homes in improving maternal and perinatal outcomes [17], these homes may provide a solution to women who live far from the health facility.

### Strengths and weaknesses

The strength of this study is that it was conducted in an area that had received interventions to improve MNCH outcomes and that despite these interventions skilled birth attendance was still below the national average, hence this study documents factors that influence health facility delivery in order to better understand the context and inform design of programs to increase use of skilled care at delivery in marginalized communities.

Our study has some limitations. The *“Boma”* intervention was implemented in eight community units in Magadi District of Kajiado County but this study focuses on one community unit – Entasopia Community. Thus the geographical and cultural variation in the other seven community units might not be captured, which limits the generalizability of this study results to Magadi district as a whole. Nevertheless, all the eight villages that comprise Entasopia community were at least sampled in the quantitative survey.

An additional limitation was that the interviews were done several months after delivery. There is a possibility of recall bias and thus misinterpretation of the previous birth experience on the part of the mothers who participated in this study. Efforts to address this bias included use of local language and reference to the last born child when conducting interviews.

## Conclusion

This study reports factors associated with health facility delivery among Maasai women. Those women belonging to the higher wealth quintiles, currently not married, and living near the health facility were more likely to deliver in the health facilities. High parity was negatively associated with health facility delivery.

To further increase the proportion of Maasai women delivering in health facilities and with skilled birth attendants, gender inequity and cultural practices such as lack of birth preparedness should be addressed. Transport mechanisms and waiting homes need to be established to avoid delay in reaching a health facility. The health systems also need to be functional with adequate supplies and motivated staff who work closely with the TBAs to ensure referral of pregnant mothers to the health facilities.

## Additional files

Additional file 1: Household questionnaire. The questionnaire used to collect data from women of reproductive age and also heads of the household. (DOC 114 kb)

Additional file 2: Interview Guide: Women who recently delivered at health facility. Interview guide for women who recently delivered at health facility. (DOCX 41 kb)

Additional file 3: Interview Guide: Women who recently delivered at home. Interview guide for women who recently delivered at home (DOC 59 kb)

Additional file 4: Interview Guide: Key Birth Decision Influencer. Interview guide for Key Birth Decision Influencer (DOCX 26 kb)

Additional file 5: In-depth Interview guide: Village elder. In-depth Interview Guide for Village elder (DOCX 24 kb)

Additional file 6: In-depth Interview Guide: Traditional Birth Attendants. In-depth Interview Guide with Traditional Birth Attendants (DOCX 25 kb)

Additional file 7: Focus Group Guide for Nurses, Midwives. Focus Group Guide for Nurses and Midwives (DOCX 19 kb)

Additional file 8: Focus Group Guide for Chiefs . Focus Group Guide for Chiefs (DOCX 18 kb)

Additional file 9: Focus Group Guide with CHVs. Focus Group Guide for CHVs (DOCX 19 kb)

## Abbreviations

Amref ESRC: Amref Health Africa Ethics and Scientific Review Committee (ESRC)
aOR: Adjusted odds ratio
ASAL: Arid and semi-arid areas
CHV: Community health volunteer
CI: Confidence interval
FGD: Focused group discussion
FWA: Federal wide assurance
NACOSTI: National commission for science, technology and innovation (NACOSTI)
OR: Odds ratio
TBA: Traditional birth attendant
