# Supplementary material for: Factors influencing deliveries at health facilities in a rural Maasai Community in Magadi sub-County, Kenya
Source: BMC Pregnancy Childbirth. 2018 Jan 3;18:5. doi: 10.1186/s12884-017-1632-x (PMC5751799; doi:10.1186/s12884-017-1632-x)
Supplement: Supplementary file 1 — Household questionnaire. The questionnaire used to collect data from women of reproductive age and also heads of the household. (DOC 114 kb) [file 12884_2017_1632_MOESM1_ESM.doc]

**Factors Influencing Deliveries at Health Facilities in a Rural Maasai Community in Magadi Sub-County, Kenya**

Date of interview_______________Village Code___________ HouseHold/BomaNo ___________

**SECTION I: Social economic status *(To be administered to the household head)***

**Part A : Demographic Profile for the Household Head.**

**1. Sex (Do Not Ask):**

1. Male □
2. Female □

**2. Year of Birth/ *olari leinoto*______________ Age/ *Ilarin***_______________

**3. Religion/*Enkirukoto/Kanisa***

1. Catholic □
2. Protestant □
3. Muslim □
4. Others □Specify:_____________

**4. Education level /*kaji itabakia te enkisuma***

1. None □
2. Primary □
3. Secondary □
4. College □
5. University □

**5. Marital status/*Iyama/Iyamishe***

| 1. Monogamous Marriage □ |
| --- |
| 1. Polygamous marriage □ |
| 1. Widow or widower □ |
| 1. Single □ |
| 1. Divorced or separated □ |

**6. If in a polygamous marriage, number of wives/other wives/*Kaja iyama*______**

**7. What is your present occupation/*Kaa siai iyasita*?**

1. Employed □
2. Self-employed □
3. Casual labour □
4. Unemployed □
5. Housewife □
6. Other □ (Specify)___________________

**7a. If employed, what is your profession/*Kaa siai itii*?______________________**

**7b. How many years have been in your profession/*Ilarin aja itaasishe*?____________**

**8. Tribe/*kaa abila*______________**

**Part B: Other Members of the Household**

1. **Number of members in the household/*Irara aja te nkaji*____________**
2. **Information about Household members/*Irkiliku le nkaji***

| **No** | **Name/ *Enkarna*** | **Sex**  Male=1  Female=2 | **Year of birth/Age/*Olari leinoto/ ilarin*** | **Relationship/*Enibaikinore*** | **Delivered in Last 2 Years/ *Itoishe tiatua olari obo otulusoyie***  Yes=1  No=2 | **Place delivered/*Kaji itoikio***  Hospital=1  Home=2  Others =3 (specif y) | **Birth Certificate seen/ *Enkardasi eiyonoto***  Yes=1  No=2 |
| --- | --- | --- | --- | --- | --- | --- | --- |
| **1** |  |  |  | **Self** |  |  |  |
| **2** |  |  |  |  |  |  |  |
| **3** |  |  |  |  |  |  |  |
| **4** |  |  |  |  |  |  |  |
| **5** |  |  |  |  |  |  |  |
| **6** |  |  |  |  |  |  |  |
| **7** |  |  |  |  |  |  |  |
| **8** |  |  |  |  |  |  |  |
| **9** |  |  |  |  |  |  |  |
| **10** |  |  |  |  |  |  |  |

**3. Number of school-aged children in your household/*Esiana oo nkera naitabaiti ilarin oopoyie sukul*?______________**

**3a. How many attend school/*Kaja natii sukul*?__________________**

|  |  |  |  |
| --- | --- | --- | --- |

**3b. What type of school? (tick all applicable)/*aa sukul etii?***

1. Tertiary (university/College) □
2. Private secondary school □
3. Government-aided secondary □
4. Public Primary school □
5. Private Primary school □
6. Other □Specify______________

**3c.Give reason(s) for those who don’t attend :( tick all applicable)/ *kanyoo pee etii inaitabaitie ilarin neton eitu iteru sukul?***

1. Cannot afford school fees and other school requirements □
2. Children do not want to go to school □
3. Lack of schools in the area □
4. Other □ Specify_____________________

**Part C: Household items/facilities**

**1. Which of the following items are available for use by your household?(Read the items and tick all applicable)/*Kakua masaa iyata tekuna***

1. Bicycle/*Embasikil* □
2. Motor cycle/*Otuktuk*  □
3. Car/*Enkari* □
4. Cell phone*/Esimu*  □
5. Radio/*Erendio* □
6. Television set/*Entivi* □
7. Sewing machine/*Emashini Eripare* □
8. Refrigerator / deep freeze/*Barafu* □
9. Foam mattress/*Orkondoro*  □
10. Gas/electric stove or cooker/*Enkas* □
11. Furniture suite (cushion chairs)*Sopa set* □
12. Solar panel/*Esola* □
13. Other/*Kulie* □Specify______________

**2. Buildings/structures (more than one option/material is possible,)/*Enjetare***

|  | **Type of building/structure** | **Materials used(observe and write)/ *Imasaa enjetare*** | | | **# rooms/**  ***Irgilat*** |
| --- | --- | --- | --- | --- | --- |
|  | e.g. Main house, kitchen, storage area e.t.c | **Walls/*Isunta***  Cow dung& sticks (Traditional manyatta) = 1  mud&sticks = 2  Bricks = 3  Stones = 4  Iron sheet=5  Other(specify)=6 | **Roof/*Orkaba***  Thatched with cow dung(Traditional Manyatta) = 1  Grass thatched=2  Iron sheet = 3  Tiles=4  Other(specify)=5 | **Floor/*Otiren***  Mud=1  Cement=2  Tiles=3  Other(specify)=4 |  |
| 1 |  |  |  |  |  |
| 2 |  |  |  |  |  |
| 3 |  |  |  |  |  |
| 4 |  |  |  |  |  |
| 5 |  |  |  |  |  |

**3. Does the respondent’s house have electricity (solar)/*Etii ositima enkaji?* (Observe and write)**

| 1. Yes |  | 2. No |  |
| --- | --- | --- | --- |

**4. What type of cooking fuel does your household use/*Kainyoo iyierishore*? (Tick all applicable)**

1. Firewood □ 2. Charcoal □
2. Kerosene □ 4. LPG/Gas □

5. Others: □ Specify:___________

**5. What toilet arrangements do your household have/*Kaji ipopuo joo*? (Tick only one)**

1. Pit latrine □
2. flush toilet □
3. Open fields □
4. Others: □ Specify:___________

**6. What is the main source of drinking water for your household /*Kaji itumie enkare niwokowoko*? (Tick only one)**

1. Spring/Well
2. Borehole
3. Stream/River
4. Dam/pod
5. Tank in the compound
6. Common tap
7. Tap in the compound
8. Others:  Specify:_____________

**Part D: Agricultural land for farming and livestock**

**1. Does your household own agricultural land for farming/*Iyata Olnchamba***?

| 1. Yes |  | 2. No |  |
| --- | --- | --- | --- |

**1a. If yes, how many acres/*iyakai aja*______________**

**2. Does your household own livestock/*iyatata inkishu***?

| 1. Yes |  | 2. No |  |
| --- | --- | --- | --- |

2a. If yes,

|  | **i) Type of livestock/*aabila oonkishu* (tick all that apply)** | **ii) # of livestock/*aja*?** |
| --- | --- | --- |
| 1 | Cattle/*inkishu*  □ |  |
| 2 | Goats/*Inkineji*  □ |  |
| 3 | Sheep/*Inkera*  □ |  |
| 4 | Donkeys/*Isirkon*  □ |  |

**SECTION II: Health seeking Behavior**

***(To be administered to the woman in the household who had given birth in the last 2 years)***

**Part A: Demographic Profile (skip to Part B if the woman selected is the household head)**

**1. Education level /*kaji itabakia te enkisuma***

1. None □
2. Primary □
3. Secondary □
4. College □
5. University □

**2. Religion/*Enkirukoto/Kanisa***

1. Catholic □
2. Protestant □
3. Muslim □
4. Others □Specify:_____________

**3. Marital status/*Iyama/Iyamishe***

| 1. Monogamous Marriage □ |
| --- |
| 1. Polygamous marriage □ |
| 1. Widow or widower □ |
| 1. Single □ |
| 1. Divorced or separated □ |

**4. If in a polygamous marriage, number of other wives/ *Kaja iyama* ______**

**5. What is your present occupation/*Kaa siai iyasita*?**

1. Employed □
2. Self-employed □
3. Casual labour □
4. Unemployed □
5. Housewife □
6. Other □ (Specify)___________________

**5a. If employed, what is your profession/*Kaa siai itii*?_____________________**

**5b. How many years have been in your profession/*Ilarin aja itaasishe?* ____________**

**8. Tribe/*kaa abila* _______________**

**Part B: Antenatal care and Delivery**

1. Did you see anyone for antenatal care for your most recent pregnancy/*itaduare itungana lebiotisho tenutai nibayie?*

1. Yes  2. No

**1a. If yes, whom did you see/ *tenaa eeh: kangae*? (*PROBE and tick all mentioned).***

1. Doctor
2. Nurse/Midwife
3. Traditional birth attendant
4. Community health worker
5. Other  (specify)___________________

**2. How many times did you receive antenatal care during this pregnancy/ *kaja itunganang lebiotisho litaduare?________***

**3. Where did you give birth to your most recent child/ *ketiaai itoiwuo enkerai ino nibayie*?**

1. Your home
2. Government hospital
3. Government health center
4. Government dispensary
5. Private health center or hospital
6. Other  (Specify)_________________

**4. Who assisted with the delivery of your most recent child/ *kangae nikintoiwuo enkerai nibayie*?**

1. Doctor
2. Nurse/midwife
3. Traditional birth attendant
4. Community health worker
5. Relative/friend
6. Other (Specify)_________________
7. No one

**5. IF SHE DID NOT DELIVER IN A HEALTH FACILITY: Why didn't you deliver in a health facility/ *Kainyoo piitu iisho tesipitali?***

- 1. Cost too much
  2. Facility not open
  3. Too far/ no transportation
  4. Don't trust facility/poor quality service
  5. No female provider at facility
  6. Husband/family did not allow
  7. Not customary
  8. Other (Specify)_________________

6. Did your most recent child ever receive any vaccinations to prevent him/her from getting diseases, including vaccinations received in a national immunization campaign/*Kenoto enkerai ino nibayie orkodata oibooyo moyiaritin ebaki naishooyo serikali?*(Can verify by checking vaccination card)

1. Yes  2. No  3. Don’t know

**7.** IF DELIVERED IN A HEALTH FACILITY/TENEITOISHE TE SIPITALI**: Did you experience any form of disrespect and/or abuse during your last birth*/ inoto enkisoroma tesipitali teishoi nibayie?***

1. Yes  2. No

**7a. If yes, what form of disrespect and/or abuse did you receive/ *kaa kisoroma iyimayie***?

1. Physical abuse
2. Verbal abuse
3. Non-consented care
4. Non- confidential care
5. Discrimination
6. Abandonment or withholding of care
7. Detention in the facility
8. Other (Specify)  ______________________

**8. In general, how did you feel about the care you received in your last birth/ *kanyoo induat inono te ramatata ninoto te eishoi nibayie?* [READ the possible responses]**

| Very good/ *sidai oleng* | Good/ *sidai* | Fair/ *metorono* | Bad/ *torono* | Very Bad/ *torono oleng* |
| --- | --- | --- | --- | --- |
| 1.□ | 2. □ | 3. □ | 4. □ | 5. □ |

**Name of the interviewer________________________**
