# Supplementary material for: Factors influencing deliveries at health facilities in a rural Maasai Community in Magadi sub-County, Kenya
Source: BMC Pregnancy Childbirth. 2018 Jan 3;18:5. doi: 10.1186/s12884-017-1632-x (PMC5751799; doi:10.1186/s12884-017-1632-x)
Supplement: Supplementary file 2 — Interview Guide: Women who recently delivered at health facility. Interview guide for women who recently delivered at health facility. (DOCX 41 kb) [file 12884_2017_1632_MOESM2_ESM.docx]

**Interview Guide*/enkitamayare*: Women who recently delivered at health facility*/inkituak natoishote det te sipitali*.**

**Factors Influencing Deliveries at Health Facilities in a Rural Maasai Community in Magadi Sub-County, Kenya**

Greetings

My name is ____________________________and my colleagues are _________ and ___________. We are here today on behalf of the AMREF research collaboration. Specifically, we would like to discuss your views and experiences with childbirth in the Entasopia community unit of Magadi district in Kajiado County, Kenya. This will better help us better understand the birth and delivery process in this community.

It is my hope that you will assist us in this endeavor. The way we have organized this activity is like a ‘discussion’ that will enable us to learn from you. We would encourage you to contribute as much as you can remember. There is no right or wrong answer and your views will be respected. All the discussions here will remain confidential and will only be used for research purposes.

My colleague [s] will try as much as possible to write all that we discuss but just as a back up we will also be recording the conversation, since you are likely to speak faster than we write. If this is not okay with you, you are welcome to leave now or at any time without any consequences. This discussion will take around one hour

If there are no questions, we can begin…

*Basic Demographic/Background Information/****olkilikuai lolkitamanyunoto.***

| Age***:/ilarin*** |  |
| --- | --- |
| Level of schooling:/***enitabaikia te nkisoma.*** |  |
| Marital status***:/iyama*** |  |
| How many births have you had?/**kaja eishoi ino?** |  |
| Wife occupation:/ ***esiai enkitok*** |  |
| Husband occupation:/esiai olpayian |  |
| Language(s) spoken:***/enkutuk*** |  |
| Do you have health insurance?/***iyata enkadi ebiyotisho?*** |  |

**Ice Breaker:/enkiterunoto**

1. How many children do you have?/***kaja inkera niata?***
   1. How old are they***?/ilarin maaja eata***?
   2. Where did you deliver each of your children?/***kaji itoikio nena kera?***
2. Can you tell me about your most recent pregnancy?/***tolikioki eneikununo enutai nibayie***
   1. What did you do when you found out you were pregnant***?/ kaji itaasa piyiolou ajo inuta?***

**Decision about place of birth:/engelunoto ewueji nitoikio**

1. For your most recent birth, who was involved in making the decision about place of delivery?/***kangae otengelua ewueji nitoikio?***
   1. Who initiated the conversation?/***kangae naiterua ilo rorei?***
   2. What was said?/***kainyoo etejoki?***
   3. How soon after you found out you were pregnant did you have these discussions?/***tia rishata enutai iimakitia kulo omon?***
   4. Did everyone agree on where you should deliver?/***ketonyorraitie eniiki***?/
   5. Who made the final decision on where to deliver?/ ***kangae otengelua ewueji niiki***?/
   6. Husband, yourself, mother, someone else?/***olpayian,makewon,ngutunyi,anaa kengae?***
2. Where did you deliver your last child?/***kaji itoikio enkerai nibayie***? Why did you choose to deliver there? ***/kainyoo pi tegeluo aishio teine***?
   What factors did you consider?
   Probe as necessary [NOTE: Give the person time to respond before probing; skip any probes that are already mentioned. Be careful not to make the probes leading]:
   1. **Social/:** ***Eramatare oormareita***/ Did any family members, friends, or other people in your village give you recommendations about where you should deliver?/***ekinjoo iltuganak le latia enduata ewueji niishore***? If so, what did they say?/***tenaa nejia,kaa etejo***?
   2. Did any **health providers** (i.e. doctors, nurses, community health workers, midwives, traditional birth attendants) give you advice on where to deliver?/***ekinjoo ilaasak le biotisho enduata ewueji niishore?***
   3. **Culture/olkuak:** Can you tell me about any traditions that your family practices during childbirth?***kalo kerreti esujita olmarei linyi te nkata eishoi?*** Did you think about those traditions when deciding where to deliver***?/itadamua lelo kerretin ingoru eniishore?***
   4. **Physical/elakuani:** Did you consider how far away the health facility was when deciding whether to go there or not?/***itishilayie elakuani ingoru eniishore?*** Did you think about transportation or how you would get to the place to give birth?***itadamua enikunari eniishore?***
   5. **Financial/iropiani:** Did you consider the costs of the different options? Do you know about health insurance***?/iyiolo enkadi embaare?***
   6. **Individual/makewon:** Did you think about your health or safety when deciding where to give birth?/***ingurayie biyotisho we seriani ino ingoru iweji niishore?/*** IF she had a previous birth: Did your experience with previous births influence where you decided to deliver for your most recent birth***?/amaa te eishoi nibayie ekinkuna pee imbelekeny enitoishore te eishoi nibayie?***
   7. **Knowledge/engeno:** Do you know about any other locations for delivery***?/iyiolo aiweji neishoreki?*** If so, why did you decide not to go to XX place***?/amaa*** ***neniyiolo kainyoo pee itulo kulie wejitin?***
   8. **Health System/sipitali:** What do you think about XX health facility (Probe for cleanliness, availability of medicine and equipment, staff attitudes/training, etc.)? /***kaa ijo tena weji nitoikio te biyotisho***?What do you think about home birth***?ijo a te ishoi eang?*** (Probe for her opinions about the type of care received at home, benefits and disadvantages)

**Birth Experience/enkariyiano tialo eishoi:**

1. When you went into labor, what happened?/ ***Amaa iarita engop, kaanyoo na taase***?

Probe:

- 1. Who did you contact? /***Ngae itolikio***?/(probe: traditional birth attendant, midwife, community health worker, husband, family member, other?)
  2. Did anyone go with you to the health facility?**/ketii oltungani likiriamariye sipitali?** Who***? Ngae***?What would you have done if that person wasn’t there?/ ***Kaiyoo apa iyas teneitu itum oliriamariye sipitali?***
  3. How did you get to the health facility (probe: what transportation did you use?) /***kaji ingunari sipitali?***Who arranged the transport?/ ***Kangai naigorua elototo?***
  4. Did you have any difficulties or complications during your labor experience?/***inoto engoloto iyarita engop?*** If yes, did that influence your decision to go to a health facility? /***Tenaa eee kinguna peee igelu eniishore?***
  5. Did you face any challenges in accessing care?***inoto engoloto ingoru eramatata***? If so, how did you overcome those challenges?***/amaa eyia kaji inkua pee itigilunore***?

1. Who helped you during your labor?/***kengae nikitareto iyarita engop***? What was your opinion of the care you received from those people?***kaiyoo eduata ino te eramatata ninoto te sipitali?***
2. How about the cost for the services/***kanyoo elaata eramatata?*** How much did you pay of your own money for your delivery/***kaja iropiyian emakewan nitalaa?***
   1. Did you pay for transportation/Italaa elototo? Medication/Embaata? Blood transfusion?/***Emponaroto osarge?***
   2. Did insurance help pay for your care?/***Ekitalaaka enkadi esipitali eramatata?***

**Experience of disrespect and/or abuse**

1. How do you feel about the care provided at the health facility?/ *kanyoo enduata ino teramatata ninoto tesipitali?* Were you treated in a way that made you feel humiliated or disrespected?/*Ekitaramataki tenkoitoi nidolita ajo enkisoroma ashu metii enkanyit?* If so/*ama tena eeh, what happened/kanyoo nataase?*

**Probe:**

1. Physical abuse/*Enkisoroma:* At any point during your stay at the health facility were you physically abused by the health care workers/*Tenkata nitii sipitali ekisoroma ilaasak le biotisho*? If yes*/ tena eeh*, what happened/*ka nataase*? How did you feel/suffer as a result of being physically abused*/kanyoo itoningo/kanyoo inoto te nkisoromata*?
2. Verbal abuse/*Emoroto:* At any point during your stay at the health facility were you verbally abused, shouted at or scolded by the health care workers/*ama itii sipitali* *ekisoromaki ashu ekitabuakaki irkitarni*? If yes, what happened/*amaa tenaa eeh, kanyoo* *nataase*?How did you feel/suffer as a result of being verbally abused?/*kanyoo iimayie eiimu emoroto?*
3. Non-consented care/*Eramatare nituinyoraa:* At any point during your stay at the health facility was any treatment done to you without your permission*/ama itii sipitali inoto eramatare nituinyoraa?* If yes, what procedures were done without your permission/*amaa tenaa eeh inyoo etaasaki nituinyoraa*? How did you feel as a result of the non-consented procedures you have just mentioned/*kakua iimayie teramatare nituinyoraa?*
4. Non-confidential care/*Eramaratata nemetii enkisudoroto:* At any point during your stay at the health facility were you treated in a manner that violated your confidentiality/*ama itii sipitali inoto eramatata nemetii enkisudoroto?* If yes, what happened*/amaa tenaa eeh, kainyoo nataase*? How did you feel/suffer as a result of being treated this way?/*kakua iimayie teramatare naikununo* *nejia?*
5. Discrimination/*Orkep:* At any point during your stay at the health facility were you treated differently because of your age/health status/traditional belief/level of education/economic status etc/*ama itii sipitali inoto eramatata natii orgela tenkaraki ilarin/biotisho,enkisuma,enkirukoto,imali niyata*? If yes, what happened*/amaa tenaa eeh, kainyoo nataase*? How did you feel/suffer as a result of being treated differently/*kakua iimayie toorgela*?
6. Abandonment or withholding care/aiturraa teramatare: At any point during your stay at the health facility were you left unattended by health providers when you needed care/*ama itii sipitali inoto ekitungayioki iyieu eramatata*? If yes, what happened/*aama atena eeh, kainyoo nataase*? How did you feel/suffer as a result/*kanyoo iimayie tenena?*
7. Detention in the facility/embikoto te sipitali: After delivery, were you detained at the health facility*/amaa pee indip ataisho, ekibooki tesipitali*? If yes, why were you detained*/ amaa te naa eeh , kainyoo*? For how long*/terishata nabaa*? How did you feel/suffer as a result of being detained*/kakua iimayie*?

**Recommendations/Iutarot:**

1. If you have another baby, where would you want to deliver/*amaa tenitum enkae kerai kaji iishore?* Why?/*kanyoo*?
2. Have you heard anything about the [list examples of the activities that were implemented as part of your intervention] skilled delivery, ANC, child immunization*/itoningo* *clinic oo ntuan,eishoi te sipitalu,o biotisho oo nkera*? if so, what have you heard*/tenaa ee nyoo itoningo?*
   1. Have any of these been put in practice in your village***/ketaasaki aikata nabo te kuna te nkutoto ino?***
   2. What do you think about these activities***/kainyoo enduata ino te kuna baa?***
      Probe: Whether the woman thinks they are good or not. If not, what could be changed to make them better? ***Tenemesidai kanyoo eibelekenyi?***
