# Supplementary material for: Factors influencing deliveries at health facilities in a rural Maasai Community in Magadi sub-County, Kenya
Source: BMC Pregnancy Childbirth. 2018 Jan 3;18:5. doi: 10.1186/s12884-017-1632-x (PMC5751799; doi:10.1186/s12884-017-1632-x)
Supplement: Supplementary file 3 — Interview Guide: Women who recently delivered at home. Interview guide for women who recently delivered at home (DOC 59 kb) [file 12884_2017_1632_MOESM3_ESM.doc]

**Interview Guide/*enkitamayare:* Women who recently delivered at home/*intomonok natoishote det tiang’.***

**Factors Influencing Deliveries at Health Facilities in a Rural Maasai Community in Magadi Sub-County, Kenya**

Greetings

My name is ____________________________and my colleagues are _________ and ___________. We are here today on behalf of the AMREF research collaboration. Specifically, we would like to discuss your views and experiences with childbirth in the Entasopia community unit of Magadi district in Kajiado County, Kenya. This will better help us better understand the birth and delivery process in this community.

It is my hope that you will assist us in this endeavor. The way we have organized this activity is like a ‘discussion’ that will enable us to learn from you. We would encourage you to contribute as much as you can remember. There is no right or wrong answer and your views will be respected. All the discussions here will remain confidential and will only be used for research purposes.

My colleague [s] will try as much as possible to write all that we discuss but just as a back up we will also be recording the conversation, since you are likely to speak faster than we write. If this is not okay with you, you are welcome to leave now or at any time without any consequences. This discussion will take around one hour

If there are no questions, we can begin…

*Basic Demographic/Background Information/****olkilikuai lolkitamanyunoto****.*

| Age:/***ilarin*** |  |
| --- | --- |
| Level of schooling***:/enitabaikia te nkisoma.*** |  |
| Marital status:/iyama |  |
| How many births have you had***?/kaja eishoi ino?*** |  |
| Wife occupation:/ ***esiai enkitok*** |  |
| Husband occupation***:/esiai olpayian*** |  |
| Language(s) spoken***:/inkutukie*** |  |
| Do you have health insurance***?/iyata enkadi ebiyotisho?*** |  |

**Ice Breaker*:/enkiterunoto***

1. How many children do you have***?/kaja inkera niata?***
   1. How old are they***?/ilarin maaja eata***?
   2. Where did you deliver each of your children***?/kaji itoikio***
2. Can you tell me about your most recent pregnancy?/***tolikioki eneikununo enutai edet?***
   1. What did you do when you found out you were pregnant***?/ kaji itaasa piyiolou ajo inuta?***

**Decision about place of birth:/*engelunoto ewueji nitoikio***

1. For your most recent birth, who was involved in making the decision about place of delivery***?/kangae otegelua ewueji nitoikio?***
   1. Who initiated the conversation?/***kangae naiterua ilo rorei?***
   2. What was said?/***kainyoo etejoki?***
   3. How soon after you found out you were pregnant did you have these discussions?/ ***Katiatua ingolong’i aja itunute enaa peiponunu aimaki kuna baa?***
   4. Did everyone agree on where you should deliver?/***Itonyorrakinote apa pookin ewueji niiki?***/Who made the final decision on where to deliver***?/ kangae otegelua ewueji niiki?***/Husband, yourself, mother, someone else?/***olpayian,kayie ,ngutunyi,anaa kang’ae?***
2. Where did you deliver your last child?/***kaji itoikio enkerai nibayie?*** Why did you choose to deliver there? ***/kainyoo pi tegeluo aiki ine?*** What factors did you consider? ***Kakua baa apa ing’urra peitegelutua ine weuji?***Probe as necessary [NOTE: Give the person time to respond before probing; skip any probes that are already mentioned. Be careful not to make the probes leading]:
   1. **Social/:** ***Eramatare oormareita/*** Did any family members, friends, or other people in your village give you recommendations about where you should deliver***?/ekinjoo iltuganak le latia enduata ewueji niishore?*** If so, what did they say?/***tenaa nejia,kaa etejo?***
   2. Did any **health providers** (i.e. doctors, nurses, community health workers, midwives, traditional birth attendants) give you advice on where to deliver?/***ekinjoo ilaasak le biotisho enduata ewueji niishore?***
   3. **Culture*/olkuak*:** Can you tell me about any traditions that your family practices during childbirth?/***kalo kerreti esujita olmarei linyi te nkata eishoi?*** Did you think about those traditions when deciding where to deliver***?/itadamua lelo kerretin ingoru eniishore?***
   4. **Physical/*elakuani*:** Did you consider how far away the health facility was when deciding whether to go there or not***?/itishilayie elakuani ingoru eniishore?*** Did you think about transportation or how you would get to the place to give birth?***itadamua eninkunari eniishore?***
   5. **Financial/*iropiyiani:*** Did you consider the costs of the different options? Do you know about health insurance***?/iyiolo enkadi ebiotisho?***
   6. **Individual/*makewon*:** Did you think about your health or safety when deciding where to give birth?/***itadamu****a* ***apa biotisho arashu eseriani ino igira agelu ewueji niishore?***/ IF she had a previous birth: Did your experience with previous births influence where you decided to deliver for your most recent birth***?/amaa imbaa niimayie too ishoritin e dukuya ekinguna mbelekenya ewueji nitoikio teishoi nibayie?***
   7. **Knowledge/*engeno*:** Do you know about any other locations for delivery?/***iyiolo aiweji neishoreki?*** If so, why did you decide not to go to XX place***?/amaa etuinejia, kanyoo peitugelu?***
   8. **Health System/*sipitali*:** What do you think about XX health facility (Probe for cleanliness, availability of medicine and equipment, staff attitudes/training, etc.)? ***/kaa enduata ino tialo ina sipitali enaa te usafi, endumoto orkeek, eramatata oo laramatak?*** What do you think about home birth?/***kanyioo enduata ino te ishoi e ang?*** (Probe for her opinions about the type of care received at home, benefits and disadvantages)

**Birth Experience/enkariyiano tialo eishoi:**

1. When you went into labor, what happened***?/ Amaa iarita engop, kaanyoo nataase?***

Probe:

- 1. Who did you contact? ***/Ngae itolikio?***/(probe: traditional birth attendant, midwife, community health worker, husband, family member, other?)

1. Who helped you during your labor?**/*kengae nikitareto iyarita engop?*** What was your opinion of the care you received from those people?***kaiyoo eduata ino te eramatata ninoto tiang’?***
2. How about the cost for the services? How much did you pay of your own money for your delivery? ***Kebaa iropiyiani nitaala piitum ina retoto?***
   1. Did you pay for a traditional birth attendant? Medication? ***Italaa apa enkaitoyioni nikintoiwuo? Oo embaata?***

**Recommendations:**

1. If you have another baby, where would you want to deliver? Why? ***Amaa taata eningil atum ae kerai kai iyieu ninauraki? Kanyioo?***
2. Have you heard anything about the [list examples of the activities that were implemented as part of your intervention]?/***Itoning’o imbaa naipirrita orkordata loontauan arashu orkordata loo nkera?*** If so, what have you heard? / ***Amaa tenaa nejia kakua baa itoning’o?***
   1. Have any of these been put in practice in your village? ***Kakua tiatua nena eesitae tenkutoto inyi?***
   2. What do you think about these activities? ***Kanyioo enduata ino te kuna baa?***
      Probe: Whether the woman thinks they are good or not. If not, what could be changed to make them better?
