# Supplementary material for: Factors influencing deliveries at health facilities in a rural Maasai Community in Magadi sub-County, Kenya
Source: BMC Pregnancy Childbirth. 2018 Jan 3;18:5. doi: 10.1186/s12884-017-1632-x (PMC5751799; doi:10.1186/s12884-017-1632-x)
Supplement: Supplementary file 4 — Interview Guide: Key Birth Decision Influencer. Interview guide for Key Birth Decision Influencer (DOCX 26 kb) [file 12884_2017_1632_MOESM4_ESM.docx]

**Interview Guide: Key Birth Decision Influencer (husband/mother/mother-in-law)**

**Factors Influencing Deliveries at Health Facilities in a Rural Maasai Community in Magadi Sub-County, Kenya**

Greetings

My name is ____________________________and my colleagues are _________ and ___________. We are here today on behalf of the AMREF research collaboration. Specifically, we would like to discuss your views and experiences with childbirth in the Entasopia community unit of Magadi district in Kajiado County, Kenya. This will better help us better understand the birth and delivery process in this community.

It is my hope that you will assist us in this endeavor. The way we have organized this activity is like a ‘discussion’ that will enable us to learn from you. We would encourage you to contribute as much as you can remember. There is no right or wrong answer and your views will be respected. All the discussions here will remain confidential and will only be used for research purposes.

My colleague [s] will try as much as possible to write all that we discuss but just as a back up we will also be recording the conversation, since you are likely to speak faster than we write. If this is not okay with you, you are welcome to leave now or at any time without any consequences. This discussion will take around one hour

If there are no questions, we can begin…

*Basic Demographic/Background Information:*

| Age: ***Ilarin:*** |  |
| --- | --- |
| Level of Schooling: ***Enkisuma ino:*** |  |
| Language(s) spoken: ***Inkutukie:*** |  |
| Occupation: ***Esiai:*** |  |

**Ice Breaker: *Enkiterunoto:***

1. Can you tell me about your wife’s/daughter’s/daughter-in-law’s last pregnancy. What happened when you found out she was pregnant?

Tolikioki siyie eneikununo eishoi nabayie enkitok ino/entito ino/enkitok olayioni lino. Amaa peeyiolou ajo entuaa kaa itaasa?

**Decision about place of birth: *Engelunoto e wueji naishore:***

1. Where did your wife/daughter give birth to the child? At home or in a health facility?

Katiaji apa etoishe entomononi ino/entito ino? Tiang enaa te sipitali?

- 1. Probe: IF delivery was at a health facility: Which facility?

Tenguton: amaa tenaa te sipitali, tia nabo?

- 1. Does your family use the health facility for other things, such as immunizations, ANC, etc.?

Keloshi ormarei lino sipitali tenkaraki kulie baa ebiotisho naijo orkordata loo nkera o ntuaan?

1. Can you tell me how the decision was made about where your wife/daughter/daughter-in-law gave birth?

Probe:

Tolikioki ajo kaikunaki pee etegeluaki enetoishore enkitok ino/entito ino/enkitok olayioni lino?

Tenguton:

- 1. Who initiated the conversation?

Kang’ae naiterua ina kiroroto?

- 1. What was said?

Kaa etejoki?

- 1. Did these discussions take place before she went into labor?

Amaa inaa kiroroto ketaase apa eton eitu esayusayu?

- 1. Did everyone agree on where she should deliver?

Ketonyorrakinote apa lelo pooki ooti ina kiroroto ewueji neishore?

- 1. Who made the final decision on where to deliver? Husband, woman herself, mother/mother-in-law?

***Kangae natumuta eweji neiki?***

1. Why did you and your wife/daughter/daughter-in-law choose to deliver at [XX place]? What factors did you consider?

Kanyioo pee itengelua iyie oo enkitok ino/entito ino/ enkitok olayioni lino pee eishore (XX ewueji)?

Kakua baa apa ing’urra?

Probe as necessary [NOTE: Give the person time to respond before probing; skip any probes that are already mentioned. Be careful not to make the probes leading]:

Tenguton: (Njoo oloikilikuani engata metushuku eton eitu ilo dukuya aikilikuanu kulie kikilikuanat).

- 1. **Social:** Did any family members or friends or other people in your village give you recommendations about where your wife/daughter/daughter-in-law should deliver? If so, what did they say?

Eramatare ormarei: Ketii oltungani lormarei, olchore arashu likae tungani likinjoo eutaroto ewueji neidim enkitok ino/entito ino//enkitok olayioni lino atoishore? Kaa utaroto apa kinchoo?

- 1. Did any **health providers** (i.e. doctors, nurses, community health workers, midwives, traditional birth attendants) give your wife/daughter/daughter-in-law advice on where to deliver?

Ketii ilangeni le biotisho enaa (orkitarri, inkaitoyiok, enkitarri, ilaasak le biotisho) oishoo eutaroto ewueji neishore enkitok ino/entito ino/enkitok olayioni lino?

- 1. **Culture:** Can you tell me about any traditions that your family practices during childbirth? Did you think about those traditions when deciding where to deliver?

Orkuak: Ketii orkerreti osuji tormarei lino tenkata einoto enkerai? Itadamu apa nena baa tinakata ingira agelu ewuaji neishore entomoni?

- 1. **Physical:** Did you consider how far away the health facility was when deciding whether to go there or not? Did you think about transportation or how your wife/daughter/daughter-in-law would get to the place to give birth?

Elakuani: itadamua apa elakuani esipitali eton eitu igelu ajo piilo arashu peemilo aishore? Oo entumoto e ngarri arashu eneikunari enkitok ino/enkerai ino/enkitok olayioni lino peebaya enelo aishore?

- 1. **Financial:** Did you consider the costs of the different options? Do you know about health insurance?

Iropiyiani: ingurra apa entumoto oo ropiyiani tengelunoto ewueji neishore? Itoning aka esiai e laata e biotisho?

- 1. **Individual:** Did the health condition of your wife/daughter/daughter-in-law play a role in where she gave birth? How? Did you have any concerns about safety?

Makewuan: Amaa bitisho e nkitok/entito/enkitok olayioni lino ketolimuo ewueji neiki? Tia oitoi?Inguraitie apa imbaa eseriani?

- 1. **Knowledge:** Do you know about any other locations for delivery? If so, why were they not chosen?

Engeno: iyiolo kulie wuejiting neidimi aatoishore? Amaa enetii kanyioo peetegeluaki?

- 1. **Health System:** What do you think about XX health facility (Probe for cleanliness, availability of medicine and equipment, staff attitudes/training, etc.)? What do you think about home birth? (Probe for their opinions about the type of care received at home, benefits and disadvantages)

Imbaa e biotisho: kakua damunot iyata tialo XX sipitalini (Tenguton: biotisho, entumoto oo rkeek oo masaa e mbaare, osim lorkitarrini/enkarriyino)? Kaa duata iyata tialo eishoi e ang? (Tenguton: toonduat enye naipirrta oramatei otum tiang isidan oo ntorrok)

**Birth Experience: *mbaa niimayie iisho?***

1. When your wife/daughter/daughter-in-law went into labor, what happened?

Amaa pee esayusayu enkitok ino/entito olayioni lino kanyio nataase?

- 1. Did everything happen the way you planned or expected?

Ketaasate imbaa enaa yieno ino?

- 1. Did you face any challenges in accessing care? If so, how did you overcome those challenges?

Inoto apa engoloto tentumoto e eramatata?amaa tenetii, kai inkuna pee igilunore?

1. What was your opinion of the care she received during the delivery?

Kanyioo enduata ino tialo eramatata natangamua tenkata eishoi?

1. Can you tell us about the costs for the delivery? Did your wife/daughter/daughter-in-law pay for any health services? Traditional birth attendant? Transportation? Medication?

Tolimu engolon telaata oo ropiyiani naimayie tengata eishoi? Ketalaa apa enkitok ino/entito/enkitok olayioani lino iropiyiani pengamu eramatata e sipitali? Enakaitoyioni? Elototo e sipitali? Embaata?

**Recommendations: *Endaata:***

Where would you want your wife/daughter/daughter-in-law to have her next birth? Why?

Kai eiki enkitok/entito/ enkitok olayioni lino teishoi nasuju? Kanyoo?

Have you heard anything about the [list examples of the activities that were implemented as part of your intervention]? If so, what have you heard?

Itoningo akata imbaa naipirrita (tolimu imbaa natasaki naipirrita ena utaroto)? Amaa tenetii, kanyioo itoning,o?

1. Have any of these been put in practice in your village? What do you think about these activities?
   Probe: Whether the person thinks they are good or not. If not, what could be changed to make them better?

Amaa tekuna mbaa ketii inaatipiki inkias? Kanyioo eduata ino tialo kuna mbaa?

Tenguton: tenaa keita oloikilikuani nena mbaa aa kesidan arashu katorrok. Tenaa katorrok kanyioo idimi ataas peitameloki?
