# Supplementary material for: Factors influencing deliveries at health facilities in a rural Maasai Community in Magadi sub-County, Kenya
Source: BMC Pregnancy Childbirth. 2018 Jan 3;18:5. doi: 10.1186/s12884-017-1632-x (PMC5751799; doi:10.1186/s12884-017-1632-x)
Supplement: Supplementary file 5 — In-depth Interview guide: Village elder. In-depth Interview Guide for Village elder (DOCX 24 kb) [file 12884_2017_1632_MOESM5_ESM.docx]

**In-depth Interview Guide_Village elder**

**Factors Influencing Deliveries at Health Facilities in a Rural Maasai Community in Magadi Sub-County, Kenya**

Greetings

My name is ____________________________and my colleagues are _________ and ___________. We are here today on behalf of the AMREF research collaboration. Specifically, we would like to discuss your views and experiences with childbirth in the Entasopia community unit of Magadi district in Kajiado County, Kenya. This will better help us better understand the birth and delivery process in this community.

It is my hope that you will assist us in this endeavor. The way we have organized this activity is like a ‘discussion’ that will enable us to learn from you. We would encourage you to contribute as much as you can remember. There is no right or wrong answer and your views will be respected. All the discussions here will remain confidential and will only be used for research purposes.

My colleague [s] will try as much as possible to write all that we discuss but just as a back up we will also be recording the conversation, since you are likely to speak faster than we write. If this is not okay with you, you are welcome to leave now or at any time without any consequences. This discussion will take around one hour

If there are no questions, we can begin…

*Basic Demographic/Background Information*

| Age: ***Ilarin:*** |  |
| --- | --- |
| Level of Schooling: ***Enkisuma ino*** |  |
| Language(s) spoken: ***Enkutuk*** |  |
| How many years have you lived in the community?  ***Kaja ilarin litobiko tena murua?*** |  |

**Ice Breaker: *Enkiterunoto:***

Can you tell me a bit about yourself and your role as a community leader/elder?
Probe: What are your responsibilities in the community?

***(I’d like to talk to you about your practices around pregnancy and childbirth in your community)***

***Tolikioki ajo ira nga’e oo esiai niasita ira olarikoni lena murua?***

Teng’uton: kanyioo esiai ino enaa olarikoni tena murua?

(Maimaki imbaa naipirrita enutai oo eishoi te murua ino).

Decision about Place of Birth: Enyorraroto e wueji neishoreki:

1. Where do women in your community deliver their babies?

**Probe**: Where are the closest facilities that support deliveries?

***Kaji oshi eiki ntomonok tena murua?***

***Tenkuton: kaji etii sipitali nataana naiki ntomonok?***

1. What factors influence where a woman gives birth?

**Probe as necessary***[NOTE: Give the person time to respond before probing; skip any probes that are already mentioned. Be careful not to make the probes leading]:*

***Kakwa baa naalimu ewueji neiki entomononi?***

- 1. **Social**: How do family and friends play a role in the decision about where to give birth, if any? How about other people such as the midwife or TBA? Do you have any influence over where women deliver? Do women ever consult you about the decision?

***Eramatare oormareita:*** *kaa tia oitoi eidim ormarei, olchoreta aategelu ewueji neiki entomononi? Oo rkulie tungana lijo nkaitoiyok/sipitali? Iata yie inkidimata peyie ilimu eneiki ntomonok? Ekinkilikwan oshi intomonok tialo ina gelunoto?*

- 1. **Culture**: Are there any cultural traditions around birth that you think might influence where a woman delivers her baby? Any religious traditions?

***Orkwaak:*** *ketii imbaa orkuak naipirta eishoi naidim aatolimu eneishore entomononi? Oo tenkoitoi orkuak le Kanisa?*

- 1. **Health System**: How do you think women’s previous birth experiences play a role in where they give birth for subsequent children?

***Biotisho:*** *Amaa te nduata ino kai iko imbaa naimatie intomonok tekulie ishoritin peelimu ewueji neigil aishore?*

- 1. **Physical:** Do women think about how far they live from a health facility when deciding where to give birth? What about access to transportation? If women in your community go to a health facility, how do they get there?

***Elakuani:*** *Kedamu oshi intomonok elakuani ewueji neishore? Oo entumoto oo ng’arrin? Amaa tenaa kepu intomonok te murau ino sipitali, kai ikunarri?*

- 1. **Financial:** Does poverty or access to money play a role in the decision? How?

***Iropiyiani:*** *kelimu aisinanisho arashu entumoto oo oropiyiani ewueji neishore entomononi? Tia oitoi?*

- 1. **Individual:**What is the pregnant woman’s role in the decision-making about where to give birth?Does her health influence the decision? How?

***Entomononi makeon:*** *Kelimu ninye entomononi ewueji neidimayu pee eishore? Kelimu biotisho enye? Tia oitoi?*

- 1. **Knowledge**: In your opinion, how do people in this community view the health facility(ies) [mentioned in Question 2 above]?

***Eng’eno:*** *amaa te nduata ino kakua duaat eeta olorere lena murua tialo sipitalini?*

1. What are your opinions about women giving birth?

***Kakwa induaat inono eipirta eishoi oo ntomonok?***

At home?

Tiang?

At a health facility?

Tesipitali?

At [add in any other places she may give birth]

Ae wueji…

BOMA Intervention: Tayiolo-Project e Josphine

**Explain the Boma intervention**

Did you play a role in implementing the project? If so, what role did you play? How did it affect your workload?Who else was involved in the implementation?

***Iata apa erishata teina project? Kaa apa esiai ino? Kangae likae otii apa ina siai? Ketoponari apa esiai ino tenkaraki ina project?***

Can you tell me about your experience with the BOMA program?Probe for what they thought about the program, what went well, what was challenging?

***Kainyoo apa kintayiolo ina arashu engoloto niimaye?***

What, if any, impact do you think the program has had?

***Kainyoo (tenetii) esidano, arashu entorroni eina program?***

Recommendations: / Iutarot:

1. Have you heard about any of the [list examples of the activities that were implemented as part of your intervention]?

***Toning’o nabo e kuna baa:***

***a). Clinic oo ntuaan?***

***b). Eishoi te sipitali?***

***c). Biotisho oo nkera kutiti?***

- 1. Have any of these been put in practice in your village?

***Ketaasaki aikata kuna te nkutoto ino?***

- 1. What do you think about these activities?
     Probe: Whether the person thinks they are good or not. If not, what could be changed to make them better?

***Kaa enduata ino te kuna baa?***

1. The government has been trying to encourage more women to deliver their babies at health facilities.

***Kenyokita serikali aikok intomonok metoisho tesipitali:***

1. What do you think about free maternity services? Probe for What works and what are the challenges?

***Kaa enduata ino te ishoi e pesho te sipitali? Kainyoo naidimayu naa kainyoo nagol tena ishoi e sipitali nemelaki?***

1. What else could be done to help more women from your community use health facilities for delivery?

***Kainyoo ae naidimi aataas naret ntomonok peyie epuo aisho te sipitali?***

Thanks you for your participation.

***ASHE TENKATA INO!***
