# Supplementary material for: Factors influencing deliveries at health facilities in a rural Maasai Community in Magadi sub-County, Kenya
Source: BMC Pregnancy Childbirth. 2018 Jan 3;18:5. doi: 10.1186/s12884-017-1632-x (PMC5751799; doi:10.1186/s12884-017-1632-x)
Supplement: Supplementary file 6 — In-depth Interview Guide: Traditional Birth Attendants. In-depth Interview Guide with Traditional Birth Attendants (DOCX 25 kb) [file 12884_2017_1632_MOESM6_ESM.docx]

In-depth Interview Guide for Traditional Birth Attendants

**Factors Influencing Deliveries at Health Facilities in a Rural Maasai Community in Magadi Sub-County, Kenya**

Greetings

My name is ____________________________and my colleagues are _________ and ___________. We are here today on behalf of the AMREF research collaboration. Specifically, we would like to discuss your views and experiences with childbirth in the Entasopia community unit of Magadi district in Kajiado County, Kenya. This will better help us better understand the birth and delivery process in this community.

It is my hope that you will assist us in this endeavor. The way we have organized this activity is like a ‘discussion’ that will enable us to learn from you. We would encourage you to contribute as much as you can remember. There is no right or wrong answer and your views will be respected. All the discussions here will remain confidential and will only be used for research purposes.

My colleague [s] will try as much as possible to write all that we discuss but just as a back up we will also be recording the conversation, since you are likely to speak faster than we write. If this is not okay with you, you are welcome to leave now or at any time without any consequences. This discussion will take around one hour

If there are no questions, we can begin…

*Basic Demographic/Background Information (to be asked at the end of interview)*

| Age: ***Ilarin:*** |  |
| --- | --- |
| Level of Schooling: ***Enkisuma ino:*** |  |
| Language(s) spoken: ***Enkutuk*** |  |
| How many years have you lived in the community?  ***Kaja ilarin litobiko tena murua?*** |  |

**Ice Breaker:**

1. Can you briefly describe your career as a midwife/TBA and what led you to your current position? Probe:

***Tolikioki te ndorropo esiai ino anaa enkaitoyioni, naa kaja inkunakine ina rishata?***

***Tenguton:***

- 1. How and where did you learn to be a midwife/TBA?

***Kaja inkuna peyie inoto enkarriyiano ena siai?***

- 1. How long have you worked as a midwife? How long in this village?

***Kebaa enkata nitaasishe anaa enkaitoyioni? Oo tena murua?***

Can you describe this community’s traditions around giving birth?

Tolikioki orkwaak lena murua tialo eishoi?

Can you walk me through a typical day/week as a midwife/TBA in this community?

***Imaki siye enkolong ino anaa enkaitoyioni…***

What are your typical duties?

***Kainyoo esiai ino?***

What sort of information do you give women during their pregnancy?

***Kakwa kiliku oshi incho intuaan?***

Are there any fees for your services? (probe: how much do women/patients have to pay)

***Kekilaki oshi toki? Tenguton: Enabaa?***

1. Are you currently working with any of the midwives or nurses from the [nearby health facility]?

***Ketii oshi taata inkaitoyiok e sipitali niasishosho tenebo?***
If no: Why?
***Amaa tenemetii, kainyoo?***

If yes:

***Tenetii;***

1. Can you tell me about when, why and how you started working with them?
   Probe: Was relationship voluntary or ‘forced’? If the choice was voluntary, probe as to why she decided to form a working relationship.

***Tolikioki ajo anu, kainyoo naa tiaa oitoi interutua aasisho te nebo?***

***Tenguton: Teyeunoto ino anaa?***

1. Can you describe how you interact with the health facility nurses and midwives during ANC and during delivery?
   Probe: What does each person do? Who makes the decisions about care? Are there any challenges to working with village midwives/TBAs/CHWs? Any benefits?

***Kaja inko te niasisho te nebo oo nkitarrini tenkata orkordata loo ntuaan o tenkata eishoi?***

***Tenguton: kaa esiai enye? Kangae eitegeli eramatare enkerai/entomononi?***

***Ketii engoloto teniasishosho o lelo tunganak? (CHWs/midwives/TBAs)***

***Ketii esidano naje?***

**Decision about Place of Birth: */ Engelunoto e wueji Neishoreki:***

1. Where do women in your community deliver their babies?

***Kaji eiki intomonok tena kutoto?***

1. Who is usually involved in making the decision where to give birth and why?
   Probe: mother, mother-in-law, elder, health worker/TBA, religious leader, aunt, husband, facility health providers, friends, others?

Probe:

***Kang’ae nagelu ewueji neikini naa kainyoo?***

- 1. Who has the final say on where she will deliver?

***Kang’ae namut ewueji neikini?***

- 1. At what point in a woman’s pregnancy does the decision about where the mother will give birth get made?

***Ka tiaa rishata enutai elimuni eneiki entomoni?***

- 1. What is your role in helping families decide?

1. What factors influence a woman and her family’s decision where to give birth?

Probe as necessary [NOTE: Give the person time to respond before probing; skip any probes that are already mentioned. Be careful not to make the probes leading]:

***Kakwa baa naalimu ewueji neiki entomononi?***

- 1. **Culture:** Are there any cultural traditions around birth that you think might influence where a woman delivers her baby? Any religious traditions?

***Eramatare oormareita:*** *kaa tia oitoi eidim ormarei, olchoreta aategelu ewueji neiki entomononi? Oo rkulie tungana lijo nkaitoiwuok/sipitali? Iata yie inkidimata peyie ilimu eneiki ntomonok? Ekinkilikwan oshi intomonok tialo ina gelunoto?*

- 1. **Health System:** How do you think women’s previous birth experiences play a role in where they give birth for subsequent children?

***Orkwaak:*** *ketii imbaa orkuak naipirrita eishoi naidim aatolimu eneishore entomononi? Oo tenkoi orkuak le Kanisa?*

- 1. **Physical:** Do women think about how far they live from a health facility when deciding where to give birth? What about access to transportation? If women in your community go to a health facility, how do they get there?

***Biotisho:*** *Amaa te duata ino kai iko imbaa naimatie intomonok tekulie ishoritin peelimu ewueji neigil aishore?*

- 1. **Financial:** Does poverty or access to money play a role in the decision? How?

***Elakuani:*** *Kedamu oshi intomonok elakuani ewueji neishore? Oo entumoto oo ng’arrin? Amaa tenaa kepu intomonok te murau ino sipitali, kai ikunarri?*

- 1. **Individual:** Does the woman’s health influence the decision? How?

***Iropiyiani:*** *kelimu aisinanisho arashu entumoto oo oropiyiani ewueji neishore entomononi? Tia oitoi?*

- 1. **Knowledge:** In your opinion, how do people in this community view the health facility [mentioned in question 7]? Do women know that they can use the health facility for delivery?

***Entomononi makeon:*** *Kelimu ninye entomononi ewueji neidimayu pee eishore? Kelimu biotisho enye? Tia oitoi?*

1. What are your opinions about women giving birth at home? At a health facility? At [add in any other places she may give birth]

***Kainyoo esiai ino teniret ilmareita metegelu eneiki.?***

**Birth Experience/ *Iniimayie te siai enkaitoyioni***

When a woman you are taking care of goes into labor, what do you do?

***Amaa tenening enkoshoke entomononi ning’orita, kaa ias?***

When you do deliveries yourself in the village, what happens?

(Probe for her role in the birth, who else is involved)

***Amaa te nintoyiu openy tiang, kainyoo naasa?***

Do you ever take women in labor to the health facility? Why or why not?
(Probe for the challenges and possible solutions)

***Irik oshi ntomonok naisho sipitali?***

**Recommendations: / *Iutarot:***

1. Have you heard about any of the [list examples of the activities that were implemented as part of your intervention]?

***Toning’o nabo e kuna baa:***

***a). Clinic oo ntuaan?***

***b). Eishoi te sipitali?***

***c). Biotisho oo nkera kutiti?***

1. Have any of these been put in practice in this area?

***Ketaasaki aikata kuna te nkutoto ino?***

1. What do you think about these activities?
   Probe: Whether the person thinks they are good or not. If not, what could be changed to make them better?

***Kaa enduata ino te kuna baa?***

**ASHE OLENG TE NKATA INO**
