# Supplementary material for: Factors influencing deliveries at health facilities in a rural Maasai Community in Magadi sub-County, Kenya
Source: BMC Pregnancy Childbirth. 2018 Jan 3;18:5. doi: 10.1186/s12884-017-1632-x (PMC5751799; doi:10.1186/s12884-017-1632-x)
Supplement: Supplementary file 7 — Focus Group Guide for Nurses, Midwives. Focus Group Guide for Nurses and Midwives (DOCX 19 kb) [file 12884_2017_1632_MOESM7_ESM.docx]

Focus Group Guide for Nurses and Midwives

**Factors Influencing Deliveries at Health Facilities in a Rural Maasai Community in Magadi Sub-County, Kenya**

Greetings

My name is ____________________________and my colleagues are _________ and ___________. We are here today on behalf of the AMREF research collaboration. Specifically, we would like to discuss your views and experiences with childbirth in the Entasopia community unit of Magadi district in Kajiado County, Kenya. This will better help us better understand the birth and delivery process in this community.

It is my hope that you will assist us in this endeavor. The way we have organized this activity is like a ‘discussion’ that will enable us to learn from you. We would encourage you to contribute as much as you can remember. There is no right or wrong answer and your views will be respected. All the discussions here will remain confidential and will only be used for research purposes.

My colleague [s] will try as much as possible to write all that we discuss but just as a back up we will also be recording the conversation, since you are likely to speak faster than we write. If this is not okay with you, you are welcome to leave now or at any time without any consequences. This discussion will take around one hour

If there are no questions, we can begin…

*Basic Demographic/Background Information*

| Age: |  |
| --- | --- |
| Level of Schooling: |  |
| Language(s) spoken: |  |
| How many years have you lived in the community? |  |

**Ice Breaker:**

1. Can you briefly describe your career as a midwife/nurse and what led you to your current position? Probe:
   1. How and where did you learn to be a midwife/nurse?
   2. How long have you worked as a midwife/nurse? How long in this facility?
2. Can you describe this community’s traditions around giving birth?

Can you walk me through a typical day/week as a midwife/nurse in this facility?

What are your typical duties?

What sort of information do you give women during ANC?

Are there any fees for your services? (probe: how much do women have to pay)

1. Are you currently working with any of the midwives, TBAs, or CHWs from the nearby villages? If no: Why?
   If yes:
   1. Can you tell me about when, why and how you started working with them?
      Probe: Was relationship voluntary or ‘forced’? If the choice was voluntary, probe as to why she decided to form a working relationship.
   2. Can you describe how you interact with the village midwives, TBAs or other community health workers during ANC and during delivery?

Probe: What does each person do? Who makes the decisions about care? Are there any challenges to working with village midwives/TBAs/CHWs? Any benefits?

**Decision about Place of Birth:**

Where do women in your community deliver their babies?

1. Who is usually involved in making the decision where to give birth and why?
   Probe: mother, mother-in-law, elder, health worker/TBA, religious leader, aunt, husband, facility health providers, others?

Probe:

- 1. Who has the final say on where she will deliver?
  2. At what point in a woman’s pregnancy does the decision about where the mother will give birth get made?
  3. What is your role in helping families decide?

1. What factors influence where a woman gives birth?

Probe as necessary [NOTE: Give the person time to respond before probing; skip any probes that are already mentioned. Be careful not to make the probes leading]:

Culture: Are there any cultural traditions around birth that you think might influence where a woman delivers her baby? Any religious traditions?

- 1. **Health System**: How do you think women’s previous birth experiences play a role in where they give birth for subsequent children?
  2. **Physical:** Do women think about how far they live from a health facility when deciding where to give birth? What about access to transportation? If women in your community go to a health facility, how do they get there?
  3. **Financial:** Does poverty or access to money play a role in the decision? How?
  4. **Individual:** Does the woman’s health influence the decision? How?
  5. **Knowledge:** In your opinion, how do people in this community view the health facility(ies) [mentioned in question 7 above]? Do women know that they can use the health facility for delivery?

1. What are your opinions about women giving birth at home? At a health facility? At [add in any other places she may give birth]

**Birth Experience:**

When a woman in labor comes to this facility, what do you do? (Probe for her role in the birth, who else is involved, what is good and bad about giving birth in the facility)

**Recommendations:**

1. What challenges do you think women face when trying to go to a health facility for delivery?
   Probe:
   1. Do some women face more challenges than others (probe older/younger, cultural group, wealth, language, etc.)
   2. Do women with health insurance get the same services as the women who pay for the services?
2. Have you heard about any of the [list examples of the activities that were implemented as part of your intervention]?
   1. Have any of these been put in practice in this area?
   2. What do you think about these activities?
      Probe: Whether the person thinks they are good or not. If not, what could be changed to make them better?
3. The [insert country name] government has been trying to encourage more women to deliver their babies at health facilities. What do you think could be done to help more women use health facilities for delivery?
