# Supplementary material for: Factors influencing deliveries at health facilities in a rural Maasai Community in Magadi sub-County, Kenya
Source: BMC Pregnancy Childbirth. 2018 Jan 3;18:5. doi: 10.1186/s12884-017-1632-x (PMC5751799; doi:10.1186/s12884-017-1632-x)
Supplement: Supplementary file 8 — Focus Group Guide for Chiefs . Focus Group Guide for Chiefs (DOCX 18 kb) [file 12884_2017_1632_MOESM8_ESM.docx]

Focus Group Discussion Guide for Chiefs

**Factors Influencing Deliveries at Health Facilities in a Rural Maasai Community in Magadi Sub-County, Kenya**

Greetings

My name is ____________________________and my colleagues are _________ and ___________. We are here today on behalf of the AMREF research collaboration. Specifically, we would like to discuss your views and experiences with childbirth in the Entasopia community unit of Magadi district in Kajiado County, Kenya. This will better help us better understand the birth and delivery process in this community.

It is my hope that you will assist us in this endeavor. The way we have organized this activity is like a ‘discussion’ that will enable us to learn from you. We would encourage you to contribute as much as you can remember. There is no right or wrong answer and your views will be respected. All the discussions here will remain confidential and will only be used for research purposes.

My colleague [s] will try as much as possible to write all that we discuss but just as a back up we will also be recording the conversation, since you are likely to speak faster than we write. If this is not okay with you, you are welcome to leave now or at any time without any consequences. This discussion will take around one hour

If there are no questions, we can begin…

*Basic Demographic/Background Information (to be filled at the end of the interview)*

| Age: |  |
| --- | --- |
| Level of Schooling: |  |
| Language(s) spoken: |  |
| How many years have you lived in the community? |  |

**Ice Breaker:**

Can each of you tell me a bit about yourself and your role as a chief or assistant chief?
Probe: What are your responsibilities in the community?

***(I’d like to talk to you about your practices around pregnancy and childbirth in your community)***

**A) Decision about Place of Birth:**

1. Where do women in your community deliver their babies?
   1. **Probe**: Where are the closest facilities that support deliveries?
2. What factors influence where a woman gives birth?

**Probe as necessary***[NOTE: Give the person time to respond before probing; skip any probes that are already mentioned. Be careful not to make the probes leading]:*

- 1. **Social**: How do family and friends play a role in the decision about where to give birth, if any? How about other people such as the midwife or TBA? Do chiefs and assistantchiefs have any influence over where women deliver? Do women ever consult you about the decision?
  2. **Culture**: Are there any cultural traditions around birth that you think might influence where a woman delivers her baby? Any religious traditions?
  3. **Health System**: How do you think women’s previous birth experiences play a role in where they give birth for subsequent children?
  4. **Physical:** Do women think about how far they live from a health facility when deciding where to give birth? What about access to transportation? If women in your community go to a health facility, how do they get there?
  5. **Financial:** Does poverty or access to money play a role in the decision? How?
  6. **Individual:**What is the pregnant woman’s role in the decision-making about where to give birth?Does her health influence the decision? How?
  7. **Knowledge:**In your opinion, how do people in this community view the health facility(ies) [mentioned in Question 2 above]?

1. What are your opinions about women giving birth

At home?

At a health facility?

At [add in any other places she may give birth]

BOMA Intervention:

**Explain the Boma intervention**

Did any of you play a role in implementing the project? If so, what role did you play? How did it affect your workload? Who else was involved in the implementation?

For those involved in implementing the project, can you tell me about your experience with the BOMA program? Probe for what they thought about the program, what went well, what was challenging

What, if any, impact do you think the program has had?

Recommendations:

1. Have you heard about any of the [list examples of the activities that were implemented as part of your intervention]?
   1. Have any of these been put in practice in your village?
   2. What do you think about these activities?
      Probe: Whether they thinks they are good or not. If not, what could be changed to make them better?
2. The government has been trying to encourage more women to deliver their babies at health facilities.

What do you think about free maternity services? Probe for what works and what are the challenges

What else could be done to help more women from your community use health facilities for delivery?

Thanks you for your participation.
